# The Effect of a Supratrochlear Spur on Patellofemoral Cartilage in Patients With Trochlear Dysplasia

**DOI:** 10.1177/03635465251323806

**Published:** 2025-03-04

**Authors:** Jakob Ackermann, Berfin Caliskan, Martin Hartmann, Lazaros Vlachopoulos, Sandro F. Fucentese

**Affiliations:** † Department of Orthopedics, Balgrist University Hospital, University of Zurich, Zurich, Switzerland; ‡ Department of Orthopedics and Trauma Surgery, Lucerne Cantonal Hospital, Lucerne, Switzerland; Investigation performed at Balgrist University Hospital, University of Zurich, Zurich, Switzerland

**Keywords:** patellofemoral instability, supratrochlear spur, bump, cartilage, trochlear dysplasia, MRI

## Abstract

**Background:**

The presence of a supratrochlear spur has been shown to influence outcomes in patients with trochlear dysplasia and is thought to accelerate cartilage wear. However, the current literature does not provide an evidence-based threshold for a relevant supratrochlear spur height.

**Purpose/Hypothesis:**

The purpose of this study was to establish a clinically significant supratrochlear spur height associated with patellofemoral chondral damage to guide surgeons in surgical decision-making. It was hypothesized that a supratrochlear spur negatively affects patellofemoral articular cartilage, with large spurs having the greatest effect.

**Study Design:**

Case series; Level of evidence, 4.

**Methods:**

This study evaluated 363 knees with trochlear dysplasia that were scheduled to undergo surgery for the treatment of patellar instability at a single institution. All patients underwent preoperative true lateral radiography and magnetic resonance imaging (MRI). There were 2 independent reviewers who analyzed the supratrochlear spur height by measuring the distance between a tangent at the anterior femoral cortex and the most prominent point of the trochlea on sagittal MRI as well as other common patellofemoral parameters. All MRI scans were assessed for full-thickness cartilage lesions.

**Results:**

Of the included 363 knees, 91 (25.1%) showed full-thickness cartilage defects on the patella, while 21 (5.8%) had full-thickness trochlear cartilage damage. Patellar defects were significantly correlated with patient’s age (*r* = 0.237; *P* < .001), body mass index (*r* = 0.148; *P* = .005), and supratrochlear spur height (*r* = 0.196; *P* < .001). Trochlear defects were significantly associated with patient’s age (*r* = 0.160; *P* = .002), patellar tilt (*r* = 0.202; *P* < .001), tibial tubercle–trochlear groove distance (*r* = 0.128; *P* = .014), and supratrochlear spur height (*r* = 0.151; *P* < .004). Trochlear dysplasia types B and D showed a trend toward a higher prevalence in patellar defects (*P* = .082), while they were significantly associated with a higher prevalance of trochlear defects (*P* = .003) compared with types A and C. Knees with patellar (5.1 ± 2.0 vs 4.3 ± 1.7 mm, respectively; *P* = .001) and trochlear (5.3 ± 2.1 vs 4.4 ± 1.8 mm, respectively; *P* = .015) cartilage defects had a significantly larger supratrochlear spur height than knees without patellar and trochlear defects. A supratrochlear spur height ≥6 mm had adjusted odds ratios of 2.7 (95% CI, 1.6-4.5; *P* < .001) and 3.4 (95% CI, 1.3-8.8; *P* = .014) for developing patellar and trochlear cartilage damage, respectively.

**Conclusion:**

A supratrochlear spur was significantly associated with patellofemoral cartilage damage. Large supratrochlear spurs demonstrated a substantially increased risk of developing patellofemoral cartilage damage.

Trochlear dysplasia is commonly observed in patients with patellar instability and substantially raises the risk of recurrent patellar dislocations and patellofemoral chondral lesions.^2,9,18,23,26^ Dejour et al^9^ initially classified trochlear dysplasia into 3 types based on the presence of a crossing sign, the trochlear depth, and initially, the presence of a trochlear bump. They described the trochlear bump as a quantitative characteristic of trochlear dysplasia, as it indicates that the floor of the proximal trochlea is above the anterior distal femoral cortex. In their radiographic analysis, it was shown that patients with trochlear dysplasia had a mean anterior translation of the trochlear floor of 3.2 mm compared with −0.8 mm in the control group. Consequently, the authors suggested that a trochlear bump ≥3 mm should be considered as pathological.^9^ Further sharpening the classification in 1998 using computed tomography, Dejour et al^5^ described the so-called supratrochlear spur as a prominence of the trochlea on the anterior aspect of the femoral cortex, which became a cardinal sign for patients with trochlear dysplasia types B and D. It is believed that the supratrochlear spur precludes optimal patellar engagement in the proximal trochlea during early knee flexion, ultimately increasing patellar instability and articular cartilage shear forces, as the supratrochlear spur has been shown to contribute to the largest deviations in patellofemoral biomechanics.^25^ Thus, there has been increasing interest in trochleoplasty to reconstruct the dysplastic trochlea and reduce the supratrochlear spur, ultimately improving patellofemoral congruence, enhancing stability, and protecting patellofemoral cartilage.^3,8,10,27^

Initially, some authors highlighted that the presence of a supratrochlear spur greater than the pathological threshold of 3 mm (types B and D) should be seen as an indication for sulcus-deepening trochleoplasty.^6^ In fact, Fucentese et al^11^ showed that the preoperative presence of a supratrochlear spur was correlated with significantly better outcomes after trochleoplasty. Modern treatment algorithms, however, have recommended trochleoplasty only in cases with a supratrochlear spur of at least 5 mm.^7,12,24^ Yet, the current literature does not provide any evidence regarding a supratrochlear spur height threshold that reflects clinical relevance, as both published values of 3 and 5 mm have not been investigated concerning their clinical significance in patellofemoral kinematics.

Consequently, the purpose of this study was to comprehensively analyze the influence of the supratrochlear spur height on patellofemoral cartilage integrity and to establish a clinically relevant threshold to guide surgical decision-making. It was hypothesized that a supratrochlear spur negatively affects patellofemoral articular cartilage, with large spurs having the greatest effect.

## Methods

Ethical approval was granted by the local research ethics committee (No. 2020-01052), and all included patients gave their written consent.

### Patient Selection

All patients with trochlear dysplasia who were scheduled to undergo surgery for patellofemoral instability at a single institution between January 2010 and December 2020 were identified. The inclusion criteria required preoperative lateral radiography and magnetic resonance imaging (MRI; combination of 1.5 T and 3.0 T) of the index knee. All patients were diagnosed with trochlear dysplasia by a musculoskeletal radiologist and an orthopaedic surgeon using lateral radiography of the involved knee. Patients who had undergone any previous patellofemoral surgical stabilization on the affected knee were excluded.

### Clinical and Radiological Assessments

Clinical notes were reviewed to determine patient’s age, sex, and body mass index (BMI). Trochlear dysplasia was classified into types A, B, C, and D according to Dejour et al^5,9^ based on the presence of a crossing sign, a supratrochlear spur, and a double contour sign (determined by a musculoskeletal radiologist and J.A.).

**Figure 1.**
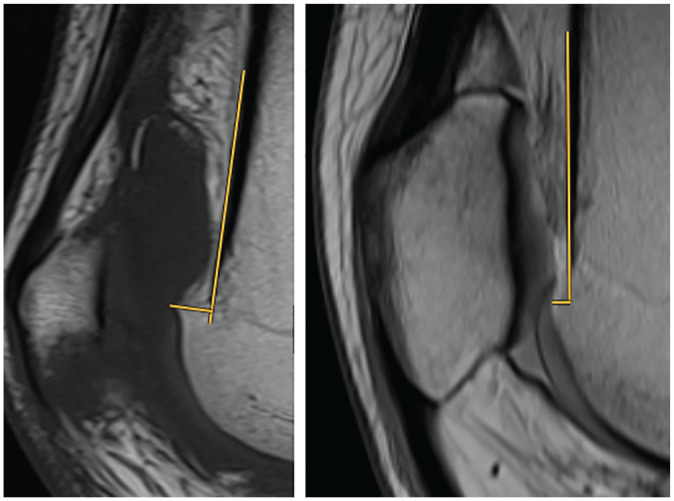
Supratrochlear spur height measurement on midsagittal magnetic resonance imaging, defined as the maximal height of the osseous trochlear floor in relation to a tangent of the anterior cortex of the distal femur. The left image shows a large supratrochlear spur height of 7.5 mm, whereas the right image shows a small anterior translation of the trochlear floor of 2.1 mm.

There were 2 independent reviewers (B.C. and M.H.) who assessed anterior translation of the trochlear floor in relation to the anterior cortex of the distal femur (supratrochlear spur height) on midsagittal MRI by measuring the maximal distance between a tangent of the anterior cortex of the distal femur and a parallel line at the supratrochlear spur tip (Figure 1).^17^ Additionally, both reviewers assessed patellar tilt,^14^ patellar height utilizing the Caton-Deschamps index (CDI),^4^ and tibial tubercle–trochlear groove (TT-TG) distance.^13^

Patellofemoral cartilage was evaluated on MRI according to previous studies and in accordance with the International Cartilage Repair Society classification system of cartilage defects.^11,20^ The current study considered only grade 4 lesions, indicating severe full-thickness cartilage damage.

All scans were read independently on a picture archiving and communication system workstation certified for clinical use (MERLIN 7.1.22; Phönix-PACS). In the event of a disagreement, a third observer (J.A.) assessed the scans to achieve a consensus.

### Statistical Analysis

Continuous variables are reported as the mean and standard deviation. The normality of distributions was examined using the Shapiro-Wilk test. Accordingly, continuous variables were analyzed with the Mann-Whitney *U* test, while the Fisher exact test and chi-square test were used for categorical variables. The intraclass correlation coefficient (ICC) with 95% confidence interval (CI) was calculated for all patellofemoral measurements including the supratrochlear spur height. The Pearson correlation coefficient was utilized to assess associations between patient’s age, sex, BMI, patellofemoral measurements, and patellofemoral cartilage defects. Lastly, multivariate logistic regression analyses were performed to calculate the odds ratio (OR) for the presence of patellofemoral cartilage lesions in relation to the supratrochlear spur height, aiming to establish a clinically meaningful threshold. All statistical analyses were performed with SPSS for Mac (Version 23.0; IBM). Significance was set at *P* < .05.

## Results

The study cohort comprised 363 knees in 330 patients (66.7% female) with a mean age of 22.5 ± 7.9 years and a mean BMI of 24.9 ± 5.4. Of the included knees, 62 (17.1%) had trochlear dysplasia type A, 122 (33.6%) had type B, 94 (25.9%) had type C, and 85 (23.4%) had type D. All knees had a mean patellar tilt of 26.4°± 10.9°, mean CDI of 1.2 ± 0.2, mean TT-TG distance of 14.3 ± 5.0 mm, and mean supratrochlear spur height of 5.1 ± 1.6 mm. The ICCs for all measurements are displayed in Table 1.

**Table 1:** Patellofemoral Measurements^*a*^

	ICC (95% CI)
Patellar tilt	0.967 (0.959-0.973)
Caton-Deschamps index	0.717 (0.652-0.770)
TT-TG distance	0.957 (0.947-0.965)
Supratrochlear spur height	0.714 (0.649-0.767)

a ICC, intraclass correlation coefficient; TT-TG, tibial tubercle–trochlear groove.

Among all knees, 91 (25.1%) had a full-thickness cartilage defect on the patella, while 21 (5.8%) had full-thickness trochlear cartilage damage. Cartilage defects on the patella were significantly correlated with increased patient age (*r* = 0.237; *P* < .001), elevated BMI (*r* = 0.148; *P* = .005), and supratrochlear spur height (*r* = 0.196; *P* < .001). Patellar tilt, CDI, TT-TG distance, and sex were not associated with patellar defects (not significant).

Cartilage defects on the trochlea were significantly correlated with increased patient age (*r* = 0.160; *P* = .002), patellar tilt (*r* = 0.202; *P* < .001), TT-TG distance (*r* = 0.128; *P* = .014), and supratrochlear spur height (*r* = 0.151; *P* < .004). Neither CDI nor sex was associated with trochlear defects (not significant).

Cartilage defects on both the patella and trochlea were more commonly seen in knees with trochlear dysplasia types B and D, with patellar defects observed in 16.1% of knees with trochlear dysplasia type A, 28.7% with type B, 20.2% with type C, and 31.8% with type D (*P* = .082). Similarly, trochlear defects were present in 0.0% of knees with type A, 6.6% with type B, 2.1% with type C, and 12.9% with type D (*P* = .003).

The ICC for the measurement of anterior translation of the trochlear floor in relation to the anterior cortex of the distal femur (supratrochlear spur height) was 0.714 (95% CI, 0.649-0.767; *P* < .001). Knees with patellar (5.1 ± 2.0 vs 4.3 ± 1.7 mm, respectively; *P* = .001) and trochlear (5.3 ± 2.1 vs 4.4 ± 1.8 mm, respectively; *P* = .015) cartilage defects had a significantly larger supratrochlear spur height than knees without patellar and trochlear defects, with an increasing incidence of cartilage defects based on the supratrochlear spur height (Table 2).

**Table 2:** Association of Supratrochlear Spur Height with Cartilage Defects^*a*^

Spur height	Knees With Patellar Cartilage Defects	Knees With Trochlear Cartilage Defects
≥3 mm (n = 255)	87 (34.1)	21 (8.2)
≥4 mm (n = 197)	74 (37.6)	17 (8.6)
≥5 mm (n = 116)	52 (44.8)	14 (12.1)
≥6 mm (n = 58)	39 (67.2)	13 (22.4)

a Data are shown as n (%).

A supratrochlear spur height ≥6 mm had ORs of 2.7 (95% CI, 1.6-4.5; *P* < .001) for developing a patellar cartilage injury and 3.4 (95% CI, 1.3-8.8; *P* = .014) for developing a trochlear cartilage injury when adjusted for age, BMI, patellar tilt and TTTG, accordingly. A supratrochlear spur height below that showed minor implications for demonstrating a patellar cartilage injury (OR, 1.6 [95% CI, 1.0-2.7]; *P* = .048) but had no significant effect on trochlear cartilage integrity (Tables 3 and 4).

**Table 3:** Odds of Developing Chondral Damage on Patella With Respect to Supratrochlear Spur Height^*a*^

Spur height	Odds Ratio (95% CI)	*P* Value
≥3 mm	1.2 (0.4-3.7)	.775
≥4 mm	1.5 (0.8-2.7)	.225
≥5 mm	1.6 (1.0-2.7)	**.048**
≥6 mm	2.7 (1.6-4.5)	**<.001**

a Analysis was adjusted for age and body mass index. Boldface values indicate a statistically significant association at *P* < .05.

**Table 4:** Odds of Developing Chondral Damage on Trochlea With Respect to Supratrochlear Spur Height^*a*^

Spur height	Odds Ratio (95% CI)	*P* Value
≥3 mm	—	—
≥4 mm	1.1 (0.3-3.5)	.880
≥5 mm	1.7 (0.6-4.5)	.308
≥6 mm	3.4 (1.3-8.8)	**.014**

a Analysis was adjusted for age, patellar tilt, and tibial tubercle–trochlear groove distance. Boldface values indicate a statistically significant association at *P* < .05.

## Discussion

The most important finding of the current study is that a supratrochlear spur was significantly associated with cartilage damage in the patellofemoral joint in patients with patellar instability. In fact, knees with a supratrochlear spur height ≥6 mm were approximately 3 times more likely to develop full-thickness cartilage defects on the patella and trochlea.

Trochlear dysplasia is a common abnormality in patients with patellar instability and patellofemoral cartilage defects; thus, the correction of trochlear dysplasia to enhance stability and decrease patellofemoral shear forces has seen increasing interest.^2,9,18,23,26^ Initially described as 1 of 3 features of trochlear dysplasia,^9^ the supratrochlear spur has sparked rising attention among orthopaedic surgeons, as it seems to significantly influence patellofemoral kinematics and may serve as a pivotal characteristic of trochlear dysplasia.^6,7,12,24,25^ In a biomechanical study, Van Haver et al^25^ investigated the effect of trochlear dysplasia on patellofemoral biomechanics with simulated trochlear deformities using 3-dimensional printing. They evaluated and compared patellofemoral kinematics, contact area, contact pressure, and stability between 4 types of trochlear dysplasia (A, B, C, and D) and a “normal” native trochlea. The authors reported that trochleae categorized as Dejour types B and D had the largest deviations in patellofemoral kinematics (smallest contact area and highest contact pressure) compared with the control group. It was concluded that the supratrochlear spur particularly seems to play a crucial role in trochlear dysplasia.^25^ In fact, the supratrochlear spur has been linked to surgical outcomes after trochleoplasty, which has resulted in the recommendation of restricting the indications for trochleoplasty to knees with trochlear dysplasia types B and D.^6-8,11,21^ Further, previous studies have revealed the association between patellofemoral cartilage deterioration and trochlear dysplasia, particularly in the presence of a supratrochlear spur, which is believed to stem from increased contact stress encountered by the patellofemoral joint during loaded flexion caused by the supratrochlear spur.^1,2,15,18,23^ Accordingly, modern treatment algorithms suggest that patients with severe trochlear dysplasia who have a supratrochlear spur of at least 5 mm should undergo surgical treatment with trochleoplasty to reduce the supratrochlear spur and optimize patellofemoral kinematics.^7,12,24^ However, the threshold of 5 mm was selected arbitrarily and has not been supported by scientific evidence yet.

While previous results have shown the effect of a supratrochlear spur on patellofemoral biomechanics,^25^ the current study is novel in quantifying the critical size of a supratrochlear spur to be clinically relevant regarding the development of patellofemoral cartilage defects. The present study does not only confirm previous findings that trochlear dysplasia types B and D, particularly, demonstrate cartilage lesions in the patellofemoral joint but also shows that the incidence of cartilage damage rose with an increase in the supratrochlear spur height. Moreover, the current results suggest that patients with a supratrochlear spur height ≥6 mm are at a 3-fold increased risk of developing full-thickness patellofemoral cartilage defects, after adjusting for other patellofemoral measurements and patient characteristics that were also significantly associated with cartilage lesions. Indeed, while only 25.1% of patellae and 5.8% of trochleae exhibited cartilage defects in the entire study cohort, these percentages increased remarkably to 67.2% and 22.4%, respectively, in knees with a supratrochlear spur height ≥6 mm. These rates align with previous studies, as Holliday et al^16^ reported an incidence of 11% for grade 4 cartilage defects in patients with patellofemoral instability, of whom 85% had signs of trochlear dysplasia. Of these, the vast majority were located on the patella.^16^ In contrast, Reinholz et al^22^ reported that 43.1% of patients with trochlear dysplasia exhibiting a supratrochlear spur (types B and D) who underwent grooveplasty or trochleoplasty also underwent concomitant cartilage repair in the patellofemoral joint, indicating the presence of full-thickness cartilage lesions in these cases.

The accentuated risk of trochlear cartilage defects with the presence of large spurs observed in the current study may be attributed to the overall lower incidence of full-thickness trochlear defects compared with patellar defects in patients with trochlear dysplasia.^2,16^ Ambra et al^2^ showed that trochlear lesions are also less often associated with anatomic risk factors such as patellar and trochlear morphology, patellar height, and quadriceps vector compared with patellar lesions. Therefore, few significant patellofemoral alterations such as large supratrochlear spurs may have a greater single effect on the trochlea than on the patella.

Consequently, this study provides evidence that large supratrochlear spurs have a clinically relevant effect on patellofemoral kinematics, which leads to cartilage deterioration. Accordingly, surgeons should be aware of the potential cartilage-protective effect of reducing large supratrochlear spurs, as the current threshold of 5 mm seems clinically reasonable for surgical reduction to optimize cartilage integrity. However, patellofemoral instability is a multifactorial condition, requiring the careful consideration of various factors, both radiological and clinical (eg, the J-sign), when making surgical decisions. Hence, future studies are warranted to investigate the influence of supratrochlear spur size on patellofemoral instability.

We also found that increased age and BMI were both correlated to patellofemoral cartilage lesions, as older patients had more time to develop cartilage defects and an elevated BMI significantly increases patellofemoral contact pressure.^19^ Interestingly, aside from the supratrochlear spur height, trochlear defects were also associated with an elevated patellar tilt and TT-TG distance in the examined patient cohort, while patellar defects did not show any association with common patellofemoral parameters. As mentioned before, this may be because of the overall lower incidence of cartilage defects on the trochlea and therefore perhaps a greater single effect of individual parameters as well as the potentially dominating effect of trochlear dysplasia itself on patellar lesions.^2^ Furthermore, we did not find a correlation between sex and patellofemoral cartilage defects in patients with trochlear dysplasia.

### Limitations

We acknowledge that there are limitations to the present study. First, this is a retrospective analysis with its inherent limitations. Thus, this study only reports an association between larger supratrochlear spurs and cartilage defects and does not prove a causative relationship. Second, cartilage lesions were only accounted for when they showed full-thickness damage. Third, reliable data regarding the number of patellar dislocations were not available. Fourth, this study did not measure cartilage lesion size. Consequently, even small lesions, which may be clinically less relevant, were included in our analysis. Fifth, the measurement for supratrochlear spur height showed only moderate reliability between both readers. Sixth, intraoperative cartilage status was not available for all patients and thus could not be correlated to MRI findings. Lastly, this study solely focused on the effect of the supratrochlear spur height on patellofemoral cartilage integrity utilizing MRI. Hence, patients did not complete patient-reported outcome measures to objectify clinical outcomes or the clinical relevance of the assessed patellofemoral cartilage lesions or patellar instability.

## Conclusion

A supratrochlear spur was significantly associated with patellofemoral cartilage damage. Large supratrochlear spurs demonstrated a substantially increased risk of developing patellofemoral cartilage damage.

## References

[1] Ali SA Helmer R Terk MR. Analysis of the patellofemoral region on MRI: association of abnormal trochlear morphology with severe cartilage defects. AJR Am J Roentgenol. 2010;194(3):721-727. 20173151 10.2214/AJR.09.3008

[2] Ambra LF Hinckel BB Arendt EA Farr J Gomoll AH. Anatomic risk factors for focal cartilage lesions in the patella and trochlea: a case-control study. Am J Sports Med. 2019;47(10):2444-2453. 31287712 10.1177/0363546519859320

[3] Balcarek P Rehn S Howells NR , et al. Results of medial patellofemoral ligament reconstruction compared with trochleoplasty plus individual extensor apparatus balancing in patellar instability caused by severe trochlear dysplasia: a systematic review and meta-analysis. Knee Surg Sports Traumatol Arthrosc. 2017;25(12):3869-3877. 27796419 10.1007/s00167-016-4365-x

[4] Caton J Deschamps G Chambat P Lerat JL Dejour H. [Patella infera: apropos of 128 cases]. Rev Chir Orthop Reparatrice Appar Mot. 1982;68(5):317-325. 6216535

[5] Dejour D Reynaud P Le Coultre B. [Patellar pain and instability: a classification (in French)]. Méd Hyg. 1998;56:1466-1471.

[6] Dejour D Saggin P. The sulcus deepening trochleoplasty: the Lyon’s procedure. Int Orthop. 2010;34(2):311-316. 20062988 10.1007/s00264-009-0933-8 PMC2899349

[7] Dejour DH Deroche E. Trochleoplasty: indications in patellar dislocation with high-grade dysplasia. Surgical technique. Orthop Traumatol Surg Res. 2022;108(1S):103160. 34863959 10.1016/j.otsr.2021.103160

[8] Dejour DH Mesnard G Giovannetti de Sanctis E . Updated treatment guidelines for patellar instability: “un menu a la carte.” J Exp Orthop. 2021;8(1):109. 34837157 10.1186/s40634-021-00430-2 PMC8626553

[9] Dejour H Walch G Nove-Josserand L Guier C. Factors of patellar instability: an anatomic radiographic study. Knee Surg Sports Traumatol Arthrosc. 1994;2(1):19-26. 7584171 10.1007/BF01552649

[10] Fucentese SF Schottle PB Pfirrmann CW Romero J. CT changes after trochleoplasty for symptomatic trochlear dysplasia. Knee Surg Sports Traumatol Arthrosc. 2007;15(2):168-174. 16786337 10.1007/s00167-006-0140-8

[11] Fucentese SF Zingg PO Schmitt J Pfirrmann CW Meyer DC Koch PP. Classification of trochlear dysplasia as predictor of clinical outcome after trochleoplasty. Knee Surg Sports Traumatol Arthrosc. 2011;19(10):1655-1661. 21302049 10.1007/s00167-011-1410-7

[12] Giovannetti de Sanctis E Mesnard G Dejour DH. Trochlear dysplasia: when and how to correct. Clin Sports Med. 2022;41(1):77-88. 34782077 10.1016/j.csm.2021.09.001

[13] Goutallier D Bernageau J Lecudonnec B. [The measurement of the tibial tuberosity: patella groove distanced technique and results (author’s transl)]. Rev Chir Orthop Reparatrice Appar Mot. 1978;64(5):423-428. 152950

[14] Grelsamer RP Bazos AN Proctor CS. Radiographic analysis of patellar tilt. J Bone Joint Surg Br. 1993;75(5):822-824. 8376449 10.1302/0301-620X.75B5.8376449

[15] Grelsamer RP Dejour D Gould J. The pathophysiology of patellofemoral arthritis. Orthop Clin North Am. 2008;39(3):269-274. 18602557 10.1016/j.ocl.2008.03.001

[16] Holliday CL Hiemstra LA Kerslake S Grant JA. Relationship between anatomical risk factors, articular cartilage lesions, and patient outcomes following medial patellofemoral ligament reconstruction. Cartilage. 2021;13(1 Suppl):993S-1001S. 10.1177/1947603519894728 PMC8808921 31876167

[17] Jian-Lussi N Pfirrmann CWA Buck FM Frauenfelder T Rosskopf AB. A novel adapted MRI-based scheme for Dejour classification of trochlear dysplasia. Skeletal Radiol. 2025;54(3):437-445. 39042200 10.1007/s00256-024-04748-7 PMC11769863

[18] Jungmann PM Tham SC Liebl H , et al. Association of trochlear dysplasia with degenerative abnormalities in the knee: data from the Osteoarthritis Initiative. Skeletal Radiol. 2013;42(10):1383-1392. 23801099 10.1007/s00256-013-1664-x PMC3757255

[19] Kim N Browning RC Lerner ZF. The effects of pediatric obesity on patellofemoral joint contact force during walking. Gait Posture. 2019;73:209-214. 31374438 10.1016/j.gaitpost.2019.07.307 PMC6707885

[20] Noyes FR Stabler CL. A system for grading articular cartilage lesions at arthroscopy. Am J Sports Med. 1989;17(4):505-513. 2675649 10.1177/036354658901700410

[21] Ntagiopoulos PG Byn P Dejour D. Midterm results of comprehensive surgical reconstruction including sulcus-deepening trochleoplasty in recurrent patellar dislocations with high-grade trochlear dysplasia. Am J Sports Med. 2013;41(5):998-1004. 23589587 10.1177/0363546513482302

[22] Reinholz AK Till SE Crowe MM , et al. Grooveplasty compared with trochleoplasty for the treatment of trochlear dysplasia in the setting of patellar instability. Arthrosc Sports Med Rehabil. 2023;5(1):e239-e247. 10.1016/j.asmr.2022.11.020 PMC9971888 36866307

[23] Stefanik JJ Roemer FW Zumwalt AC , et al. Association between measures of trochlear morphology and structural features of patellofemoral joint osteoarthritis on MRI: the MOST study. J Orthop Res. 2012;30(1):1-8. 21710542 10.1002/jor.21486 PMC3217080

[24] Trasolini NA Serino J Dandu N Yanke AB. Treatment of proximal trochlear dysplasia in the setting of patellar instability: an arthroscopic technique. Arthrosc Tech. 2021;10(10):e2253-e2258. 10.1016/j.eats.2021.05.027 PMC8556545 34754731

[25] Van Haver A De Roo K De Beule M , et al. The effect of trochlear dysplasia on patellofemoral biomechanics: a cadaveric study with simulated trochlear deformities. Am J Sports Med. 2015;43(6):1354-1361. 25740833 10.1177/0363546515572143

[26] Wierer G Krabb N Kaiser P , et al. The patellar instability probability calculator: a multivariate-based model to predict the individual risk of recurrent lateral patellar dislocation. Am J Sports Med. 2022;50(2):471-477. 35060768 10.1177/03635465211063176

[27] Zimmermann F Milinkovic DD Balcarek P. Outcomes after deepening trochleoplasty and concomitant realignment in patients with severe trochlear dysplasia with chronic patellofemoral pain: results at 2-year follow-up. Orthop J Sports Med. 2021;9(6):23259671211010404. 10.1177/23259671211010404 PMC8191084 34164556

